# Efficient and transgene-free genome editing in banana using a *REG-2* promoter–driven gene-deletion system

**DOI:** 10.1186/s43897-023-00065-0

**Published:** 2023-08-23

**Authors:** Chunhua Hu, Fan Liu, Ou Sheng, Qiaosong Yang, Tongxin Dou, Tao Dong, Chunyu Li, Huijun Gao, Weidi He, Siwen Liu, Guiming Deng, Ganjun Yi, Fangcheng Bi

**Affiliations:** 1 Institute of Fruit Tree Research, Guangdong Academy of Agricultural Sciences, Key Laboratory of South Subtropical Fruit Biology and Genetic Resource Utilization (Ministry of Agriculture and Rural Affairs), Guangdong Provincial Key Laboratory of Tropical and Subtropical Fruit Tree Research, Guangzhou, China; 2 College of Life Sciences, South China Agricultural University, Guangzhou, China; 3 Maoming Branch, Guangdong Laboratory for Lingnan Modern Agriculture, Maoming, China

## Abstract

**Supplementary Information:**

The online version contains supplementary material available at 10.1186/s43897-023-00065-0.

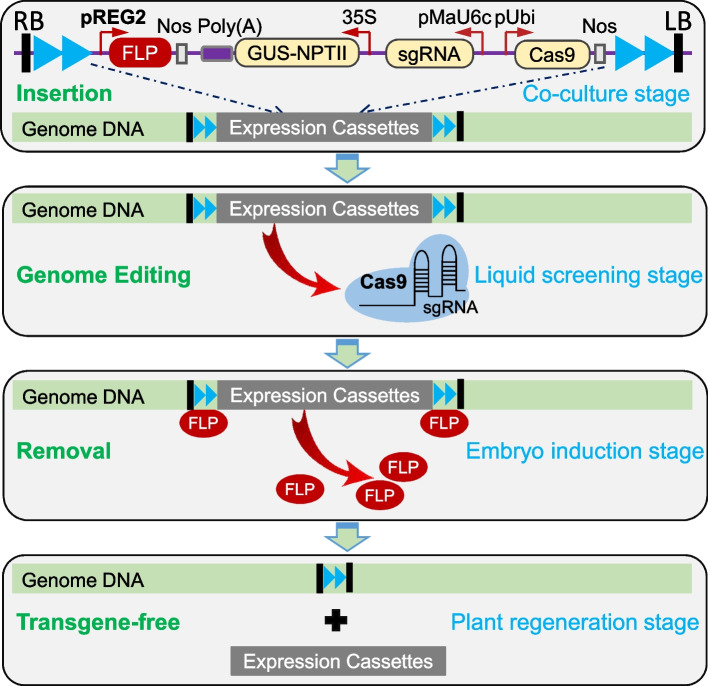

The *Streptococcus*-derived CRISPR/Cas9 system can introduce precise and predictable modifications into the plant genome to obtain the desired traits. As one of the most advanced tools for editing crop genomes, the CRISPR/Cas9 system has been expanding rapidly and has been widely applied to determine gene function and improve agronomic traits in horticultural crops such as fruits and vegetables (Ma et al. 2023).

Bananas are the most produced, traded, and consumed fruits globally, according to Food and Agriculture Organization (FAO) statistics, and thus have great global economic importance. Several factors, such as disease, pests, and drought, seriously restrict banana production (Tripathi et al. 2019). The polyploidy, heterozygosity, and sterility traits of bananas render genetic improvement using conventional methods challenging. Modern breeding tools, including genetic modification (GM) and genome editing (GE), offer great potential for agronomic trait improvement in banana.

Cultivated banana accessions generally possess a triploid genome (AAA, AAB, or ABB) and are seedless and asexually propagated. Recently, a highly efficient CRISPR/Cas9 GE system was generated in banana based *Agrobacterium*-mediated transformation of embryogenic cell suspension (ECS) cultures (Hu et al. 2017; Ntui et al. 2020), and various genetically engineered banana germplasms were created using this system (Tripathi et al. 2022; Hu et al. 2021). In these genome-edited germplasms, DNA cassettes used for genome editing were randomly integrated into the genome. However, because banana is a vegetatively propagated triploid, it is not feasible to eliminate the integrated construct from the genome by genetic segregation following crossing or selfing. Therefore, these GE germplasms may be regarded as GM plants and are thus severely restricted by complicated regulatory approval processes.

In response to these stringent regulations, scientists have attempted to directly deliver the preassembled Cas9 protein-guide RNA (gRNA) ribonucleoproteins (RNPs) into plant cells (ECS or protoplasts) (Tripathi et al. 2019). The RNPs are rapidly degraded by endogenous proteases and RNases after mutating the target sites, and hence leave no trace of foreign DNA or protein elements in plant cells. This approach has been successfully applied to various crops (Tripathi et al. 2020).

Over the years, considerable efforts have been made by our research group to generate transgene-free edited bananas using particle bombardment and polyethylene glycol (PEG)-mediated transformation with RNPs; however, these approaches showed low mutation efficiency and low repeatability. Regeneration of whole plants from protoplasts presents another obstacle. To date, an efficient and stable transgene-free CRISPR/Cas9 system in banana remains elusive. Herein, we present a strategy for producing transgene-free mutant plants by assembling the CRISPR/Cas9 system and gene-deletion system into one construct and introducing this construct into banana ECS using the *Agrobacterium*-mediated transformation protocol (Fig. 1A). The gene-deletion system that uses a site-specific recombinase (e.g., bacterial phage CRE/loxP and *Saccharomyces cerevisiae* FLP/FRT) to remove all transgenes from target sites has been extensively applied to alleviate concerns about biosafety in GM crops (Luo et al. 2007). Using this strategy, the integrated sequence is deleted after obtaining stable antibiotic-resistant ECS cultures using conventional transformation methods.

**Fig. 1:**
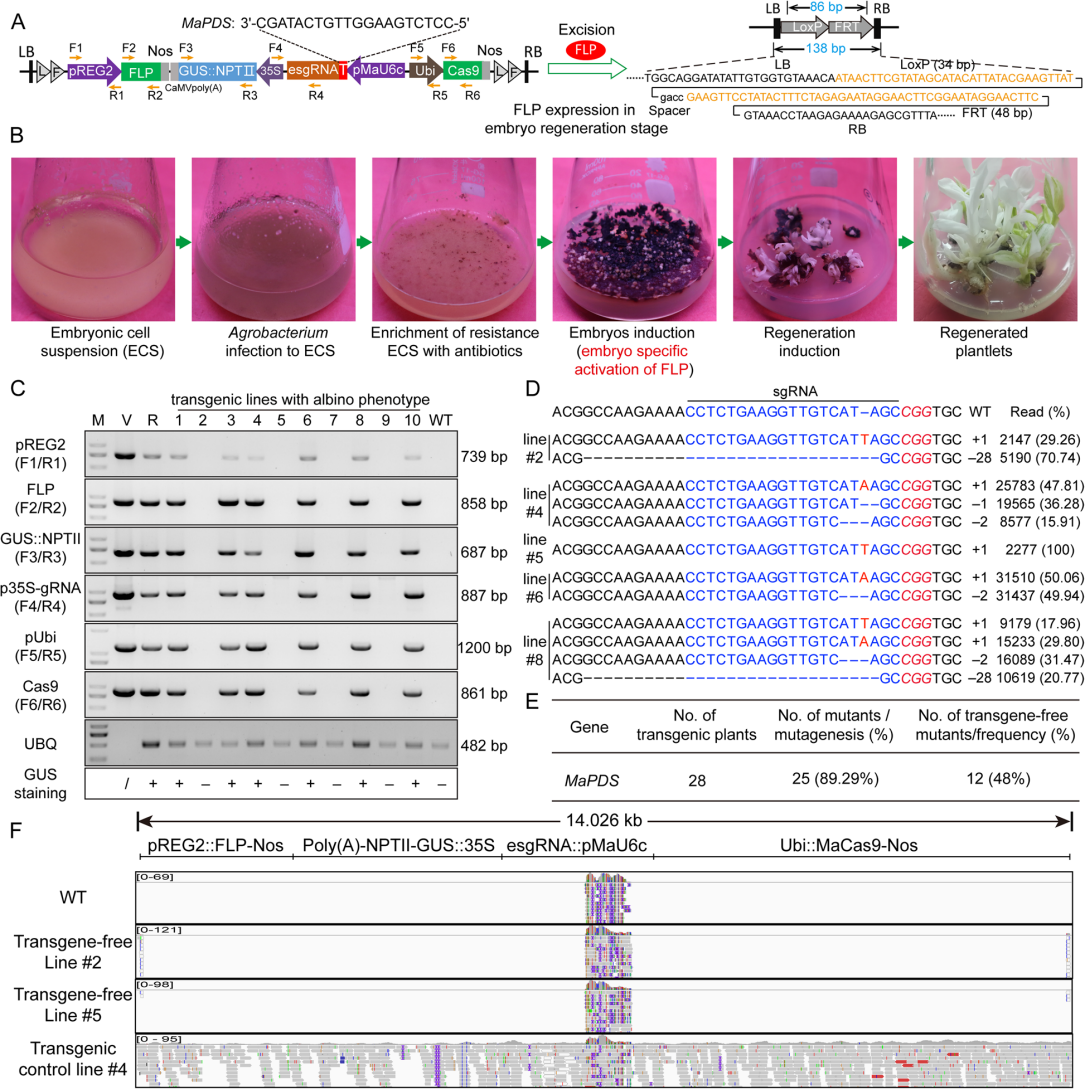
Efficient and transgene-free genome editing in banana using the gene-deletion system. **A** Strategies for generating transgene-free genome-edited plants and illustration of constructs for genome editing. Primer sets for detecting transgene-free mutants are indicated with orange arrows. **B** Procedure for transgene-free genome editing in banana. **C** Results of tests for transgene-free mutants using six primer sets in 10 representative mutant plants. M: DNA marker; V: vector plasmid; R: Kan + ; WT: wild type; /: non-determined; + : GUS staining positive; -: GUS staining negative; UBQ: ubiquitin gene. **D** Genome-edited examples of genotyping results for five mutants. **E** Gene-editing efficiencies achieved when *MaPDS* (JQ762260.1) was mutated in the T0 generation. **F** Mapping of construct DNA in the banana genome using whole genome resequencing data

To guarantee the high efficiency of mutagenesis and deletion as well as to ensure the convenience of this system, the following components were used in our approach: (1) fused *loxP-FRT* recognition sequences, which improve the excision efficiency of FLP recombinase (Luo et al. 2007); (2) banana codon-optimized Cas9 and endogenous U6c promoter, which increase the mutation efficiency in banana (Zhang et al. 2022); (3) promoter of the rice globulin *REG2* gene (AY427562.1), which offers embryo-specific deletion of the sequence between two *loxP-FRT* recognition sites without the need for extra handling (Sun et al. 1996); and (4) GUS activity provided by the GUS::NPTII expression cassette, which can be used to monitor the enrichment of antibiotic-resistant ECS and preliminarily exclude the transgene-harboring regenerated plantlets. In addition, screening of ECS cultures in antibiotic-containing liquid media can dramatically improve the recovery of transgenic plants (Dong et al. 2020). In this work, phytoene desaturase gene of *Musa acuminata* (*MaPDS*) was selected as the target gene for editing, and the mutation of *MaPDS* resulted in albino plants, a phenotype that has worked well in our previous research (Hu et al. 2017). The entire transformation process was performed as presented in Fig. 1B. The *REG-2* promoter induces the expression of *FLP* specifically during the embryo induction stage (Chong-Perez et al. 2013), which leads to the deletion of integrated sequences without extra treatment and the regenerated plants are transgene-free.

Our results indicated that approximately four-fifths of the regenerated plants were mutated at the target site and exhibited an albino phenotype (Fig. 1E). Mutation analysis with High-throughput Tracking Of Mutations (Hi-TOM) further confirmed the mutation of the target site (Fig. 1D). A PCR amplification assay was used to examine the presence of the DNA construct in the regenerated plants. Six primer sets were designed to amplify discrete regions in the genome editing plasmid, which together represented all major parts of the construct (Fig. 1A). Ten mutant lines with the albino phenotype were chosen for examination. The vector DNA and antibiotic resistant ECS cultures were considered as positive controls, and PCR amplification of the *Ubiquitin* (*Ubi*) gene fragment was used to determine the DNA quality. The results indicated that none of the vector fragments were found in the transgene-free and wild-type (WT) plants, except the *Ubi* gene fragment (lines 2, 5, 7, and 9) (Fig. 1C), and 48% of the mutated plants were transgene-free (Fig. 1E). To further verify our results, three transgenic lines and one WT plant were selected for genome resequencing. Mapping analysis indicated that the DNA construct was integrated into the genome of the transgenic control line #4, as determined by the presence of construct reads. By contrast, genomic analysis of transgene-free lines revealed no fragments of the DNA construct, except the endogenous *MaU6c* promoter and partial border sequences, as evidenced by visual analysis of the construct fragments using IGV ver. 2.15.4 (Fig. 1F). Together, these results indicated that the transgene components were successfully deleted by FLP from the plant genome.

In summary, we successfully generated a highly efficient and transgene-free genome-editing system by programmed autoexcision in banana, without extra handling. Overall, 89.29% of the transgenic plants were mutated at the target site and exhibited an albino phenotype. PCR and resequencing analyses revealed that 48% of the regenerated plants were completely transgene-free, indicating that embryo-specific autoexcision is an efficient and versatile system for removing exogenous genes from transgenic banana plants. The transgene-free system established in our study would greatly enhance fundamental research in banana and improve the banana germplasm obtained using conventional transformation protocols. Moreover, this strategy could also be expanded to other asexually propagated crops.

### Supplementary Information

**Additional file 1:** **Supplemental Table 1.** Primers used in this study.

**Additional file 2:** **Supplemental Table 2.** Summary of the whole genome re-sequencing data.

**Additional file 3:** **Supplemental Table 3.** Sequences information used in this study.

**Additional file 4.** Materials and methods used in this study.

## Data Availability

The data supporting the findings of this study are available within the article and/or its supplementary materials.
