# Supplementary material for: Efficient and transgene-free genome editing in banana using a REG-2 promoter–driven gene-deletion system
Source: Mol Hortic. 2023 Aug 23;3:16. doi: 10.1186/s43897-023-00065-0 (PMC10514973; doi:10.1186/s43897-023-00065-0)
Supplement: Supplementary file 4 — Additional file 4. Materials and methods used in this study. [file 43897_2023_65_MOESM4_ESM.docx]

Efficient and transgene-free genome editing in banana using a *REG-2* promoter–driven gene-deletion system

Chunhua Hu ^1, ☯^, Fan Liu ^1, 2, ☯^, Ou Sheng ^1^, Qiaosong Yang ^1^, Tongxin Dou ^1^, Tao Dong ^1^, Chunyu Li ^1^, Huijun Gao ^1^, Weidi He ^1^, Siwen Liu ^1^, Guiming Deng ^1^, Ganjun Yi ^1, 3, *^, Fangcheng Bi ^1, 3, *^

*^1^ Institute of Fruit Tree Research, Guangdong Academy of Agricultural Sciences, Key Laboratory of South Subtropical Fruit Biology and Genetic Resource Utilization (Ministry of Agriculture and Rural Affairs), Guangdong Provincial Key Laboratory of Tropical and Subtropical Fruit Tree Research, Guangzhou, China*

*^2^ College of Life Sciences, South China Agricultural University, Guangzhou, China*

*^3^ Maoming Branch, Guangdong Laboratory for Lingnan Modern Agriculture, Maoming, China*

^☯^ These authors contributed equally to this work

*Correspondence (Tel +86-020-87596278; email yiganjun@vip.163.com (G.Y.); Tel +86-020-38765390; email bifangcheng@gdaas.cn (F.B.))

Materials and Methods

**1. Plasmid construction**

The construction of the automatic elimination vector was started from pLF_polseed-FLP (Luo et al. 2007). Briefly, the promoter of PAB5 in pLF_polseed-FLP was replaced with *REG-2* promoter that was synthesized by GenScript Biotech Corporation (Nanjing, China) using *Bam*H I and *Sal* I sites. The pUbi::MaCas9-Nos expression cassette was amplified from our optimized genome editing vector (Zhang et al. 2022) and introduced into obtained construct using the *Bst*X I site, and then the pMaU6c::PDS-esgRNA expression cassette was cloned into the above construct using *Asc* I site. The final construct was transformed into *Agrobacterium tumefaciens* strain EHA105 for genetic transformation of embryogenic cell suspensions (ECS). The detailed sequences of pUbi::MaCas9-Nos and pMaU6c::PDS-esgRNA are available from our previous report (Zhang et al. 2022).

**2. *Agrobacterium*-mediated transformation and plant regeneration**

The *A. tumefaciens* strain EHA105 culture including genome editing construct was used for embryogenic cell suspensions (ECS) transformation of the *Musa* AA *Sucrier*, *Pisomgmas* with a previously described method (Hu et al. 2013). In brief, 1 ml packed cell volume (PCV) of ECS was added into 10 ml plasmid contained bacterial suspension with 100 μM acetosyringone. The cultures were co-cultivated on a shaker in darkness at 50 rpm, at 24°C for 3 d. After cocultivation, the infected ECS were rinsed three times with 100 ml fresh M2 medium and then were added to 40 ml M_2_S liquid medium including 50 uM Kanamycin and 100 uM Ceftriaxone to screen the putative transgenic ECS. The cultures were maintained on a shaker at 100 rpm in the dark and subcultured every 3 weeks. After 2 months, the ECS was transferred to a solid M3 medium for embryo induction and sub-cultured every 3 weeks until resistant mature somatic embryos appeared. Mature somatic embryos were cultivated in M4 medium for 1 month, and then germinated embryos were transferred to RM medium for rooting. Rooted plantlets were transferred to the greenhouse for further cultivation. Histochemical GUS assay can be used to monitor the transgenic ECS in the liquid selection stage and preliminarily determine if the integrated construct is deleted from the genome of regenerated plants.

**3. Mutation identification by High-throughput Tracking Of Mutations (Hi-TOM) analysis**

The genomic DNA is isolated from regenerated plantlets, and bout 100 ng of genomic plant DNA was used as the template for the PCR reactions. About 184 bp target regions including the target site are amplified with the following primer set 5′-CCGAGGCCTGAACTTGAG-3′ and 5′-GCAATTGGAAGGATATGATTAC-3′ in the first-round PCR. In the second-round PCR, 10-nt barcodes are added onto both ends of the forward and reverse primers, and the PCR product was used for Hi-TOM analysis. The barcodes can be used to distinguish the amplicons from different samples for His-seq analysis. The second-round PCR and the following Hi-TOM analysis are performed by Company Biorun, Inc (Wuhan, China).

**4. Transgene-free mutant determination by polymerase chain reaction (PCR)**

Normal PCR was used to examine the presence of plasmid DNAs in all regenerated plantlets. A total of six primer sets that covered all components between LB and RB borders were used to examine transgene-free mutants in plants.

**5. Whole genome sequencing and data analysis**

The 150-bp paired-end reads whole genome sequencing data were generated using the NovaSeq 6000 platform by Genedenovo. The raw reads were filtered using Fastp version 0.22.0 to remove low-quality reads. On average, more than 19.55 GB of high-quality data was generated for each sample. The high-quality paired-end short genomic reads were mapped to the reference genomes of banana (version 2.0) using BWA version 0.7.15.

**References**

Hu CH, Wei YR, Huang YH, Yi GJ. An efficient protocol for the production of chit42 transgenic Furenzhi banana (*Musa* spp. AA group) resistant to *Fusarium oxysporum*. In Vitro Cell Dev-Pl. 2013;5:584-592.

Luo KM, Duan H, Zhao DG, Zheng XL, Deng W, Chen YQ, et al. 'GM-gene-deletor': fused loxP-FRT recognition sequences dramatically improve the efficiency of FLP or CRE recombinase on transgene excision from pollen and seed of tobacco plants. Plant Biotechnol J. 2007;5:263-274.

Zhang S, Wu SP, Hu CH, Yang QS, Dong T, Sheng O, et al. Increased mutation efficiency of CRISPR/Cas9 genome editing in banana by optimized construct. PeerJ. 2022; doi:10.7717/peerj.12664.
